# Diet folate, DNA methylation and genetic polymorphisms of *MTHFR *C677T in association with the prognosis of esophageal squamous cell carcinoma

**DOI:** 10.1186/1471-2407-11-91

**Published:** 2011-03-05

**Authors:** Cheng Lu, Hui Xie, Fengliang Wang, Hongbing Shen, Jianming Wang

**Affiliations:** 1 Department of Breast, Nanjing Maternity and Child Health Hospital of Nanjing Medical University, Nanjing, PR China; 2 Department of Epidemiology and Biostatistics, School of Public Health, Nanjing Medical University, Nanjing, PR China

## Abstract

**Background:**

Folic acid may affect the development of human cancers. However, few studies have evaluated the consumption of diet folate in the prognosis of patients with esophageal squamous cell carcinoma (ESCC).

**Methods:**

One hundred and twenty five ESCC patients underwent esophagectomy between January 2005 and March 2006 in the Yangzhong People's Hospital were recruited and followed up. The effects of diet folate, aberrant DNA methylation of selected genes and methylenetetrahydrofolate reductase (*MTHFR*) C677T genetic polymorphisms on the prognosis of ESCC were evaluated by using Cox proportional hazard regression models.

**Results:**

Our analysis showed an inverse association between diet folate intake and the risk of death after esophagectomy. The median survival time was 3.06 years for low or moderate folate consumption and over 4.59 years for high folate consumption. After adjusting for potential confounders, the hazard ratios (95% confidence interval) [HRs (95% CI)] were 0.72 (0.36-1.46) for moderate and 0.39 (0.20-0.78) for high folate intake, respectively (*P *for trend = 0.007). This preventive effect was more evident in patients carrying *MTHFR *677CC genotype. No significant relation was observed between aberrant DNA methylation of *P16*, *MGMT *and *hMLH1 *gene, as well as *MTHFR *C677T genetic polymorphisms and the prognosis of ESCC.

**Conclusions:**

Our research indicated that diet folate intake may have benefits on the prognosis of ESCC after esophagectomy. From a practical viewpoint, the findings of our study help to establish practical intervention and surveillance strategies for managements of ESCC patients and can finally decrease the disease burden.

## Background

Esophageal cancer has been ranked as one of the most common cause of death from cancers worldwide [1]. In contrast with the esophageal adenocarcinoma arising from Barrett's esophagus in western countries, the major phenotype in the Asia-Pacific region is esophageal squamous cell carcinoma (ESCC) [2,3]. Possible risk factors for ESCC include cigarette smoking, alcohol drinking, hot-temperature food items, chronic mucosal irritation, and a family history of cancers [4-6]. Deficiency of nutrients, such as vitamins and micro-elements, was also found to be associated with an increased risk for ESCC, whereas a high intake of fruits and vegetables has been considered to be effective in prevention [5].

Folate is a water-soluble vitamin and naturally found in green leafy vegetables, cereals, legumes and fruits [7]. Folate is necessary for de novo synthesis of thymine, which is important in DNA synthesis, integrity and stability [8,9]. Deficiency of folate may cause defective DNA repair and chromosomal fragile site expression, leading to chromosomal breaks and micronucleus formation [7]. Furthermore, folate is the primary methyl group donor which has a central role in DNA methylation [8-10]. More and more evidence has indicated the association of folate, as well as the aberrant DNA methylation with the risk of human cancers [8,11,12]. However, the role of dietary folate in cancers is still controversial and the studies on the relation between folate intake and ESCC in Chinese population were scarce till now. Only several studies reported that dietary folate was negatively associated with the risk of endometrial cancer [13], breast cancer [14] and rectal cancer [15]. High folate intake has been found to be significantly related with better survival of patients with advanced gastric cancer who were treated with first-line fluorouracil-based chemotherapy [16]. Considering the folate pool imbalance and impaired repair mechanisms may result in DNA instability and strand breaks, we hypothesized that folate insufficiency disrupts global and specific gene methylation patterns may not only influence the susceptibility to, but also the progression of ESCC. Hence, we conducted an epidemiological study based on an esophageal cancer patient's cohort in a Chinese population.

## Methods

### Study subjects

The procedures for case recruitment were described previously [17]. Briefly, 125 ESCC patients underwent esophagectomy between January 2005 and March 2006 in the Yangzhong People's Hospital were involved in this study, which accounted for about more than 80% of cases under esophageal cancer surgery in this hospital during the same period. The eligible cases were Han Chinese living in Yangzhong for more than five years, with pathologically confirmed ESCC and informed consent. The recrudesced cancer patients or cases with the secondary ESCC from a primary cancer located elsewhere were excluded. Yangzhong is an island county on the middle of Yangtze River in southeast part of Jiangsu Province of China and is well known for the high mortality and incidence of the stomach and esophageal cancer [3]. No programs of government-mandated folate fortification of processed foods have been performed in the study site. All recruited patients were followed by professional staff of Yangzhong Cancer Research Institute by the end of September, 2009.

### Data collection

After obtaining written informed consent from all participants, trained interviewers administered the risk factor questionnaire to collect information on socio-demographic factors, tobacco smoking, alcohol drinking, and dietary habits. The food frequency questionnaire (FFQ) was used to estimate the usual dietary intake. The FFQ used in the present study was referred by NIH (National Institutes of Health, USA) and modified according to Chinese food items and cooking habits. For each food item, we collected the frequency and quantity of consumption, and then calculated daily intake by multiplying the frequency reported for the consumption of each food item by the specified portion size. Dietary intake of folate during the last 1-2 years prior to diagnosis was ascertained. Peripheral blood was collected with vacuum blood tubes. For those patients underwent surgery, tissues in the center of the cancer lesion and remote normal appearing esophagus were excised and stored in -70°C refrigerator immediately. In this study, we defined never smokers as individuals who had smoked less than100 cigarettes totally during their lifetime, and former smokers as individuals who had stopped smoking for at least 1 year at the time of diagnosis.

### Experiments

DNA was extracted by proteinase K digestion and a modified phenol-chloroform protocol. The CpG island methylation at the promoter region of *P16*, *MGMT *and *hMLH1 *was determined by methylation specific PCR (MSP) after sodium bisulfite modification of DNA [17,18]. In brief, genomic DNA was incubated with NaOH at 37°C for 10 minutes and then treated with freshly prepared hydroquinone and NaHSO_3_. Samples were incubated under mineral oil at 50°C for 16 hours. Modified DNA was purified and eluted with 50ul preheated TE solution. Modification was completed by the treatment of 5.5ul 3 M NaOH (final concentration 0.3 M) for 15-20 minutes at room temperature. DNA was precipitated by ethanol and resolved in TE. The CpG island methylation at the promoter region of *P16*, *MGMT *and *hMLH1 *genes was assessed by MSP using methylation-specific primer and relevant annealing temperature [17]. A methylation positive DNA control sample was made in vitro by SssI methylase (New England Biolabs). PCR products were loaded onto 3% gels, stained with ethidium bromide, and directly visualized under UV illumination. Methylenetetrahydrofolate reductase (*MTHFR*) C677T genetic polymorphism (rs1801133) was detected by Polymerase Chain Reaction-Restriction Fragment Length Polymorphism (PCR-RFLP). The forward primer was 'TGA AGG AGA AGG TGT CTG CGG GA' and the backward primer was 'AGG ACG GTG CGG TGA GAG TG'. PCR products were digested overnight at 37°C with restriction endonuclease *Hinf*I and then the digestive products were resolved on 3% agarose gel and visualized under UV light after staining with ethidium bromide. The variant genotype TT has two bands (175bp and 23bp). The heterozygote CT has three bands (198bp, 175bp and 23bp) while the wild genotype CC has only one 198 bp band.

### Statistical analyses

Statistical analyses were performed by using STATA 10.0 (College Station, TX, USA). Cox proportional hazards model was applied to estimate the risk by calculating hazard ratios (HRs) and the 95% confidence intervals (95% CIs). The primary death of esophageal cancer was defined as the failure event and the time of survival was the time between diagnosis and death. The cause of death was defined by specialists based on the clinical documents and reports by patient's family members. If the patient died of other causes rather than esophageal cancer, he/she was censored at the date of death. All survived patients were censored at the date of last follow-up. The diet folate intake for each food item was calculated by multiplying the weight (grams) of food intake and the folate content (per gram) referring to a national food nutrition database [19,20]. And then the sum of folate took from various foods was calculated as the total folate intake. In this study, we used the folate from fresh fruits as a representative value for the total dietary folate intake. The continuous variables of folate intake were transferred to three categories as low, moderate, and high by using tertile as the cut point. Dummy variables were used to estimate HRs for categorical variables of exposure. The differences between distinct diet folate intake categories were compared by using the Kaplan-Meier curves and Log-rank test. All tests were two-sided with a significant level of P < 0.05 based.

### Ethics consideration

This project has been approved by the IRB of the School of Public Health, Fudan University, China (IRB#05-06-0031). Ethics has been respected throughout the whole study period.

## Results

Briefly, 125 ESCC patients underwent esophagectomy between January 2005 and March 2006 in the Yangzhong People's Hospital were recruited in this study. All patients had operation within 2 weeks after diagnosis. The majority of the patients were males (64.8%) and the average age (mean ± SD) was 61.8 ± 7.0 years. Patients were followed up since the diagnosis until the end of September, 2009. Among the 125 ESCC patients, 5 were lost to follow-up due to migration. Finally, 120 patients were investigated successfully. The median time of follow-up was 3.5 years (minimum and maximum were 0.03 and 4.66 years, respectively). Fifty-eight out of them (48.33%) died of esophageal cancer during the follow-up period.

**Figure 1:**
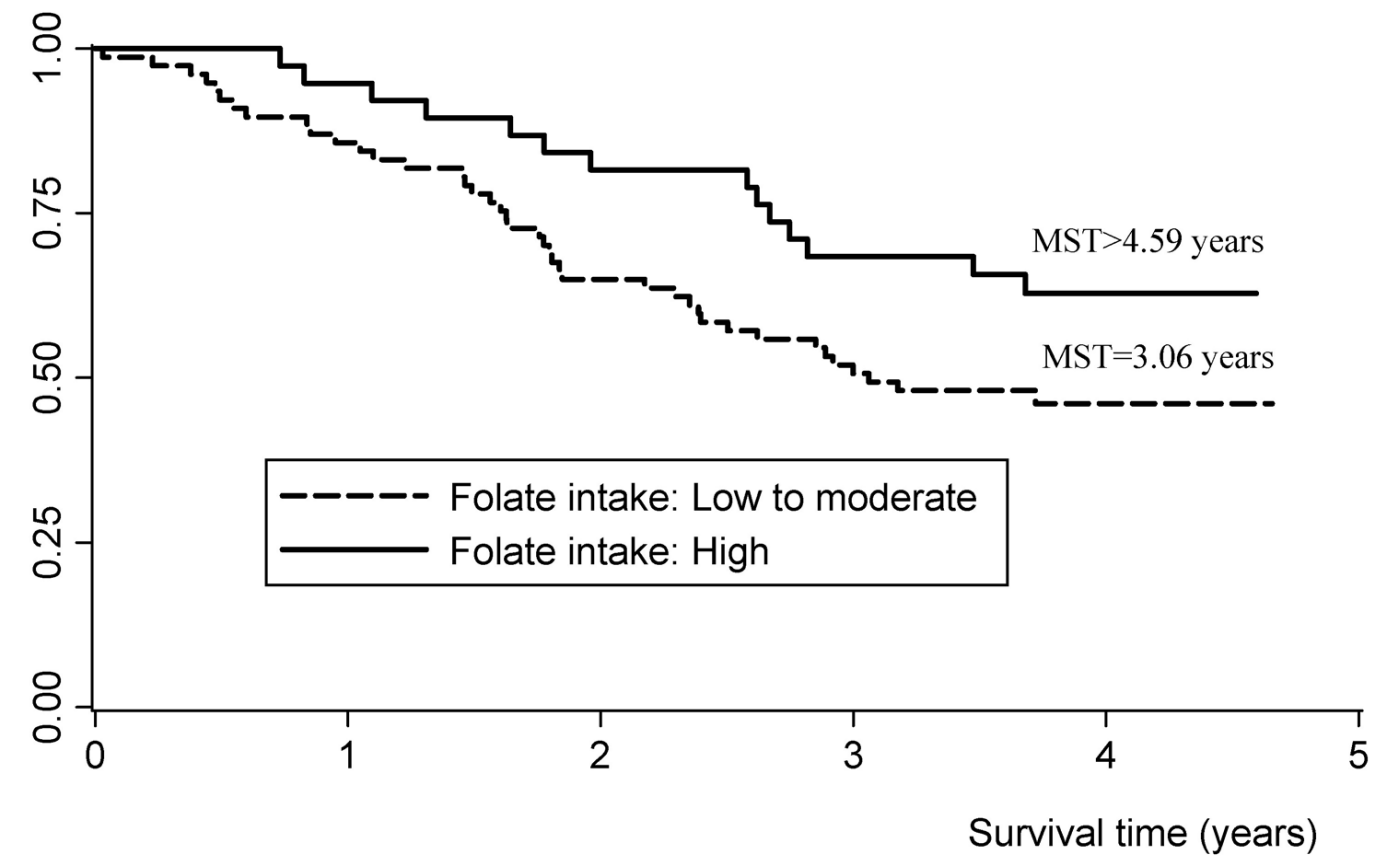
**Kaplan-Meier survival estimates for patients in the prognosis of esophageal squamous cell carcinoma**. MST: median survival time.

As shown in Table 1 no significant association was found between the prognosis of ESCC and selected demographic characteristics such as sex, age, tobacco smoking, alcohol drinking, and education level. The patients divorced or living alone had a borderline significantly increased risk of death (HR: 1.96, 95% CI: 0.99-3.88). The association between clinical characteristics and the prognosis of ESCC were shown in Table 2. Both TN and clinical stages were related to the survival time and the trend was significant too. Compared with patients at T1 stage, subjects at T2, T3 and T4 stage had elevated risks of death with the HR(95% CI) of 1.52(0.55-4.20), 3.80(1.47-9.79) and 7.65(2.4-24.32), respectively. We further explored the role of aberrant DNA methylation of *P16*, *MGMT *and *hMLH1 *genes, as well as *MTHFR *C677T genetic polymorphisms in the prognosis of ESCC, but no significant association was observed (Table 3). However, we found an inverse relation between diet folate intake and the risk of death. As shown in Figure 1 the median survival time were 3.06 years for low or moderate and more than 4.59 years for high folate consumption (log-rank test *P *= 0.063). After adjusting for potential confounders (age, sex, T stage and N stage), the HRs (95% CI) were 0.72(0.36-1.46) and 0.39(0.20-0.78) for moderate and high folate intake, respectively (*P *for trend = 0.007). After stratified by *MTHFR *C677T genotypes, the preventive effects were shown in patients carrying *MTHFR *677CC genotype with high dose of folate intake (Table 4).

**Table 1:** Association between demographic characteristics and the prognosis of ESCC

Characteristics	Total patients	Death, N(%)	HR(95% CI)^†^	*P*
Sex				
Male	79	38(48.10)	1	
Female	41	20(48.78)	1.04(0.60-1.78)	0.895
Age(years)				
< 63	61	27(44.26)	1	
≥63	59	31(52.54)	1.30(0.78-2.18)	0.320
Tobacco smoking				
Never	65	31(47.69)	1	
Ever	55	27(49.09)	1.02(0.61-1.70)	0.947
Alcohol drinking				
Never	69	33(47.83)	1	
Ever	51	25(49.02)	1.02(0.61-1.72)	0.927
Marriage				
Married	106	48(45.28)	1	
Divorced/living alone	14	10(71.43)	1.96(0.99-3.88)	0.054
Education				
Illiterate	40	20(50.00)	1	
< 6 years	46	22(47.83)	0.91(0.50-1.67)	0.759
≥6 years	34	16(47.06)	0.89(0.46-1.71)	0.719
*P *for trend				0.714

†: HR(95% CI), hazard ratio (95% confidence interval)

**Table 2:** Association between clinical characteristics and the prognosis of ESCC

Characteristics	Total patients	Death, N(%)	HR(95% CI)^†^	*P*
Site				
Upper	23	13(56.52)	1	
Middle	64	30(46.88)	0.80(0.42-1.54)	0.507
Low	33	15(45.45)	0.74(0.35-1.55)	0.424
TNM stage				
T				
1	21	5(23.81)	1	
2	44	15(34.09)	1.52(0.55-4.20)	0.414
3	47	31(65.96)	3.80(1.47-9.79)	**0.006**
4	8	7(87.50)	7.65(2.40-24.32)	**0.001**
P for trend				**< 0.001**
N				
0	62	18(29.03)	1	
1	58	40(68.97)	3.54(2.02-6.19)	**< 0.001**
M				
0	119	58(48.74)	1	
1	1	0(0)	-	-
Clinical stage				
1/2	61	20(32.79)	1	
3/4	59	38(64.41)	2.83(1.64-4.87)	**< 0.001**
Chemo-therapy				
No	114	57(50.00)	1	
Yes	6	1(16.67)	0.25(0.03-1.79)	0.167
Radio-therapy				
No	91	41(45.05)	1	
Yes	28	17(60.71)	1.45(0.83-2.56)	0.195

†: HR(95% CI), hazard ratio (95% confidence interval)

**Table 3:** Association between aberrant DNA methylation and the prognosis of ESCC

Characteristics	Total patients	Death, N(%)	HR(95% CI)^†^	P
Cancer tissue				
*P16*				
Unmethylated	14	8(57.14)	1	
Methylated	106	50(47.17)	0.67(0.32-1.41)	0.288
*MGMT*				
Unmethylated	88	43(48.86)	1	
Methylated	32	15(46.88)	1.00(0.55-1.79)	0.989
*hMLH1*				
Unmethylated	116	56(48.28)	1	
Methylated	4	2(50.00)	1.16(0.28-4.76)	0.836
Any gene				
Unmethylated	11	5(45.45)	1	
Methylated	109	53(48.62)	1.07(0.43-2.69)	0.880
Remote normal appearing tissue				
*P16*				
Unmethylated	74	36(48.65)	1	
Methylated	46	22(47.83)	1.02(0.60-1.74)	0.937
*MGMT*				
Unmethylated	107	51(47.66)	1	
Methylated	13	7(53.85)	1.35(0.61-2.97)	0.461
*hMLH1*				
Unmethylated	120	58(48.33)	-	-
Methylated	0	-	-	-
Any gene				
Unmethylated	67	32(47.76)	1	
Methylated	53	26(49.06)	1.13(0.67-1.89)	0.655
*MTHFR *C677T				
CC	35	16(45.71)	1	
CT	54	28(51.85)	1.15(0.62-2.13)	0.653
TT	31	14(45.16)	0.98(0.48-2.02)	0.964
CT+TT	85	42(49.41)	1.09(0.61-1.94)	0.770

†: HR(95% CI), hazard ratio (95% confidence interval)

**Table 4:** Diet folate intake and *MTHFR *C677T polymorphism in the prognosis of ESCC

*MTHFR *C677T	Folate intake^†^	Total patients	Death, N(%)	aHR(95% CI)*	*P*
Overall	Low	39	22(56.41)	1	
	Moderate	38	19(50.00)	0.72(0.36-1.46)	0.366
	High	38	14(36.84)	0.39(0.20-0.78)	**0.007**
	*P *for trend				**0.007**
					
CC	Low	12	7(58.33)	1	
	Moderate	12	6(50.00)	0.33(0.07-1.50)	0.152
	High	11	3(27.27)	0.14(0.03-0.76)	**0.023**
	*P *for trend				**0.025**
					
CT	Low	19	13(68.42)	1	
	Moderate	13	5(38.46)	0.62(0.18-2.09)	0.441
	High	19	8(42.11)	0.42(0.17-1.01)	0.054
	*P *for trend				0.053
					
TT	Low	8	2(25.00)	1	
	Moderate	13	8(61.54)	1.46(0.22-9.68)	0.696
	High	8	3(37.50)	0.88(0.09-8.74)	0.914
	*P *for trend				0.921

† The cut point of folate intake is tertile (μg/d). Low: <30.0; moderate: 30.0-95.4; high: ≥95.5

*: aHR(95% CI), adjusted hazard ratio (95% confidence interval); adjusted for age, sex, T stage and N stage

## Discussion

The present study indicated that ESCC patients with high folate consumption may have better survival after surgery than individuals with low diet folate intake. This protective effect was more evident in patients carrying *MTHFR *677CC genotypes. Mechanistically, this may make sense taking into account the putative effect of folate metabolism in the development and prognosis of human cancers.

Folate mediates the transfer of one-carbon moieties both in the synthesis of nucleotides necessary for DNA synthesis, replication, and repair and in DNA methylation reactions [17]. These functions may play a critical role in carcinogenesis. Evidence indicates that an abundant intake of food stuffs rich in folate conveys protection against the development of some common cancers [21]. Although various epidemiological studies have shown the inverse association between folate intake and the risk of cancers, the role of folate supplement for specific population in the carcinogenesis is still controversial [9]. In some animal studies, dietary folate deficiency can inhibit rather than enhance the development of breast cancer [22,23], which is in contrast to the observation in epidemiological studies. Some even demonstrate that an overly abundant intake of folate might instead produce a paradoxical promotion of tumorigenesis among those who harbour pre-existing, undiagnosed precancerous and cancer lesions [7,21]. Mason et al. reported a temporal association between folic acid fortification of enriched cereal grains and an increase in the incidence of colorectal cancer in the United States and Canada [24]. It has been accepted that folate possesses dual effects on carcinogenesis depending on the time and dose of intake. High consumption of folate might be a risk fact for pre-existing and cancer lesions, but could also be benefit for patients after tumour being removed.

One potential consequence of folate deficiency or abundance is the risk of modification on DNA methylation [9]. Aberrant methylation occurring in cancers includes global hypomethylation in genomic DNA as well as hypermethylation in specific gene promoters [25]. Loss of global DNA methylation may induce genomic instability and thereby promote carcinogenesis whereas promoter hypermethylation usually results in transcriptional gene inactivation [26]. In our previous study, we have reported that the aberrant CpG island hypermethylation of cancer related genes was associated with the clinical characteristics of ESCC and might be a promising biomarker for cancer diagnosis [17]. However, until now few studies have been conducted to explore the role of folate and DNA methylation in association with the prognosis of ESCC after surgery. In the present study, we didn't observe a significant association between the methylation of selected genes and the survival of patients with ESCC. It might due to the limited biomarkers we have tested and the small sample size of patients we have recruited. Another explanation was that we only tested the methylation status before surgery and patients would have changed life styles after esophagectomy.

In addition, the activity of folate metabolic enzymes, such as MTHFR, methionine synthase (MTR) and thymidylate synthase (TS) are also involved in the folate metabolic and DNA methylation process. As a key enzyme in folate metabolism, the product of MTHFR serves as the carbon donor for the methylation of homocysteine tomethionine, which is catalyzed by the enzyme MTR [27]. The *MTHFR *gene is highly polymorphic in the general population, with the most common functional variant of 677 C to T. This polymorphism results in an alanine to valine substitution, leading to a reduction in enzyme activity [28]. A pooled analysis including 725 cases and 1531 controls showed a significant association between the *MTHFR *677 TT genotype and susceptibility to esophageal cancer [28], but its effect in the prognosis of cancer patients is unclear. In the present study, we did not observe a significant independent effect of the *MTHFR *C677T polymorphism on patient's survival, which was consistent to the report by Sarbia [27]. It might due to the large variety of other factors than genetic polymorphisms which may influence the activity of the key enzymes involved in folate metabolism. For example, a study examined the folate status and *MTHFR *C677T polymorphism on genomic DNA methylation in peripheral blood mononuclear cell and found that the *MTHFR *C677T polymorphism could influence DNA methylation status through an interaction with folate status [29]. Interestingly, we found that diet folate intake had different effects on the prognosis of ESCC by different genotypes of *MTHFR *C677T. The preventive effect of folate intake was more evident in patients carrying *MTHFR *677CC genotype. This finding indicates that ESCC prognosis might be influenced by folate intake in relation to *MTHFR *C677T genotypes.

There are some limitations of this study. First, data on baseline diet were collected at enrolment, asking about consumption in the last 1-2 years prior to participants' cancer diagnosis. Studies of ESCC prognosis that rely on prediagnostic exposure information might result in substantial misclassification as patients may change life styles after diagnosis. Second, FFQ has been widely used to measure dietary folate and has been proved to be valid and economic in western countries. However, as the diet habits are significantly different between China and western countries, the application of FFQ in Chinese population is facing some difficulties. (1) The recipe in China is very complex compared to that in western countries. So it is difficult to recall and calculate the type and amount of each food item. (2) As we know, the main sources of dietary folate are fruits, vegetables, legumes, cereals, and liver [8]. The folate is susceptible to heat, pH, and oxidation. Incorrect cooking methods would influence the actual intake of folate. Estimated folate intake from total consumed food using FFQ may not accurately reflect the true intake of total dietary folate [8]. According to the traditional habits in Yangzhong County, people like to fry vegetables under higher temperature or boil it for a long time which will destroy the folate easily. It might not be appropriate to calculate folate intake by summarizing all consumed food. So, in this study, we try to use folate from fresh fruits for the indicator of total dietary folate intake. Some evidences from other studies also support it. For example, Flood [30] reported that there existed a significant correlation between fruit intake and folate intake. With the increase of fruit intake, the folate level also increased. In the present study, we used fruit folate values to estimate the total folate intake. It may not reflect the truth and more studies are needed to prove the association between high dose folate and protective effects of prognosis after esophagectomy.

## Conclusions

Our research indicated that diet folate intake may have benefits on the prognosis of ESCC after surgery. A randomized clinical trial of folate supplementation among post-esophagectomy patients is suggested to measure the effect of supplement use of folate.

## Competing interests

The authors declare that they have no competing interests.

## Authors' contributions

JW conceived the idea and implemented laboratory tests. HS and JW were involved in data collection. CL, HX, FW and JW participated in the statistical analysis and drafted the manuscript. All authors read and approved the final manuscript.

## Pre-publication history

The pre-publication history for this paper can be accessed here:

http://www.biomedcentral.com/1471-2407/11/91/prepub
